# Supplementary material for: The oldest sepioid cephalopod from the Cretaceous discovered by Digital fossil-mining with zero-shot learning AI
Source: Commun Biol. 2026 Jan 16;9:301. doi: 10.1038/s42003-026-09519-9 (PMC12929594; doi:10.1038/s42003-026-09519-9)
Supplement: Supplementary file 2 — Supplementary Information [file 42003_2026_9519_MOESM2_ESM.pdf]

Supplementary Information

The oldest sepioid cephalopod from the Cretaceous discovered by Digital fossil-mining with zero-shot learning AI

Kanta Sugiura[1], Shin Ikegami[1], Yusuke Takeda[2], Jörg Mutterlose[3], Mehmet Oguz Derin[4], Aya Kubota[5], Harufumi Nishida[6], Kazuki Tainaka[7], Takahiro Harada[4], Neil H. Landman[8], Yasuhiro Iba[1]*

[1]Department of Earth and Planetary Sciences, Hokkaido University, Sapporo 060-0810, Hokkaido, Japan.
[2]Spectroscopy and Imaging Division, Japan Synchrotron Radiation Research Institute, Sayo 679-5198, Hyogo, Japan.
[3]Department of Geosciences, Ruhr-Universität Bochum, Bochum 44801, Nordrhein-Westfalen, Germany.
[4]Morgenrot Inc., Tokyo 102-0083, Tokyo, Japan.
[5]Department of Geosciences, Osaka Metropolitan University, Sugimoto 558-8585, Osaka, Japan.
[6]Department of Biological Sciences, Chuo University, Tokyo 112-8551, Tokyo, Japan.
[7]Brain Research Institute, Niigata University, Niigata 951-8122, Niigata, Japan.
[8]American Museum of Natural History, New York 10024, NY, USA.
*Corresponding author: iba@sci.hokudai.ac.jp

Supplementary Discussion

Morphological disparity analyses of the cephalopod lower beaks

Cephalopod beaks have 46 morphological characters. Although a reliable phylogenetic tree cannot be constructed based only on fossil beaks of a single species, a large number of characters from the beaks provide robust data for morphological analyses. We therefore conducted a principal coordinates analysis using Gower's distance to validate our phylogenetic assignment of *Uluciala rotundata* gen. et sp. nov. The Gower's distance is a measure that describes the similarity between two objects in the same dataset, and is useful for multivariate statistical analyses[1]. The Gower's distances calculated for *U. rotundata* indicate that the lower beak morphology of this species is remarkably closer to that of the orders Sepiida and Sepiolida than to any other cephalopod order. The calculated values are shown in Supplementary Fig. 1, in which smaller values indicate that the taxa are morphologically more similar to *U. rotundata*. *U. rotundata* shows the lowest difference in the Gower's distances with the Sepiida in the average value at the order level (= 0.182), and to *Austrorossia bipapillata* of the Sepiolida at the species level (= 0.170). The principal coordinates analysis plot for all species examined here (Supplementary Fig. 2) indicates that *U. rotundata* is positioned among the cluster formed by the Sepiida and Sepiolida.

Four species of the order Oegopsida, which belong to genera *Bathyteuthis* and *Chtenopteryx*, are also plotted between the Sepiida and Sepiolida in the principal coordinates analysis (Supplementary Fig. 2). The taxonomic assignments of these four species have been controversially discussed due to their shared characteristics with the order Myopsida[2]. Their positions in the principal coordinates analysis plot probably reflect such deviations from ordinary oegopsids. The Gower's distances of these four species are closer to myopsids than to sepioids. The order Vampyromorpha also looks close to sepioids in the plot, but this might result from its mosaic beak morphology, partially similar to that of many other cephalopod orders. The Gower's distances from sepioids are much larger in the Vampyromorpha (average value = 0.291) than in *U. rotundata*.

These results show that the lower beak of *U. rotundata* is morphologically intermediate between that of the order Sepiida and Sepiolida, being consistent with our systematic palaeontology.

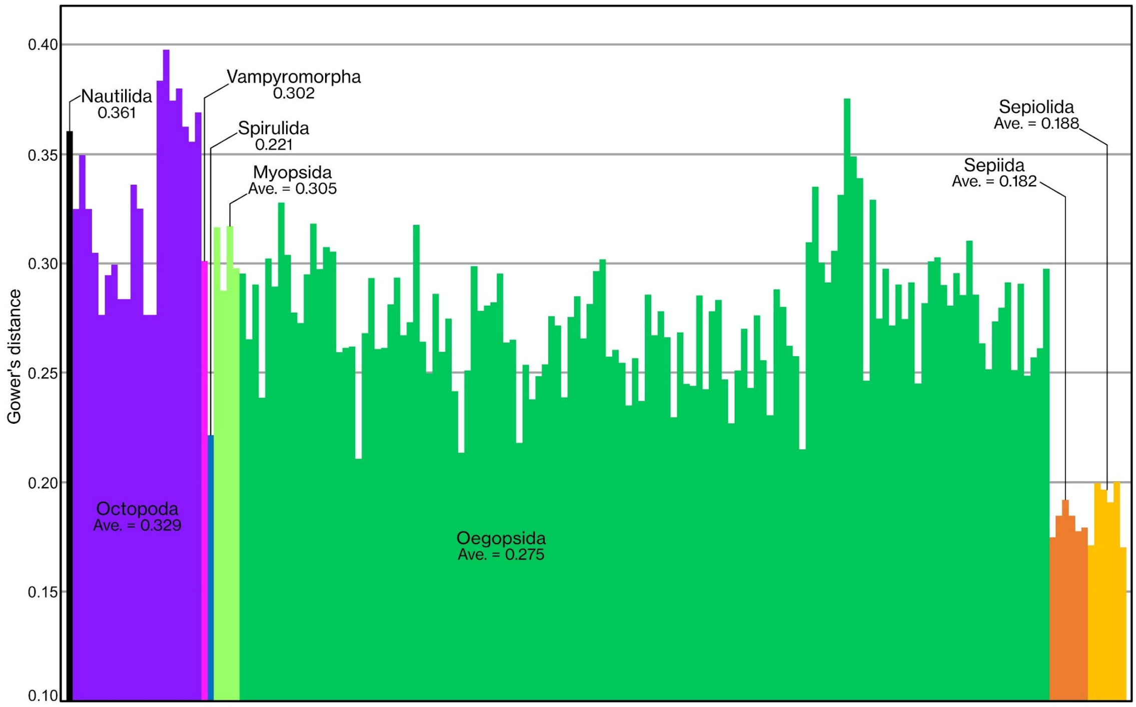

Supplementary Fig. 1. | Gower's distance from *Uluciala rotundata* gen. et sp. nov. to modern cephalopod species.

The bars show Gower's distance from *U. rotundata* to individual species of modern cephalopods based on the lower beak morphology. Smaller values indicate that the taxa are morphologically closer to *U. rotundata*. Each order of the modern cephalopods is represented by a different colour. The numbers beneath the order names are the average Gower's distance values for the respective orders.

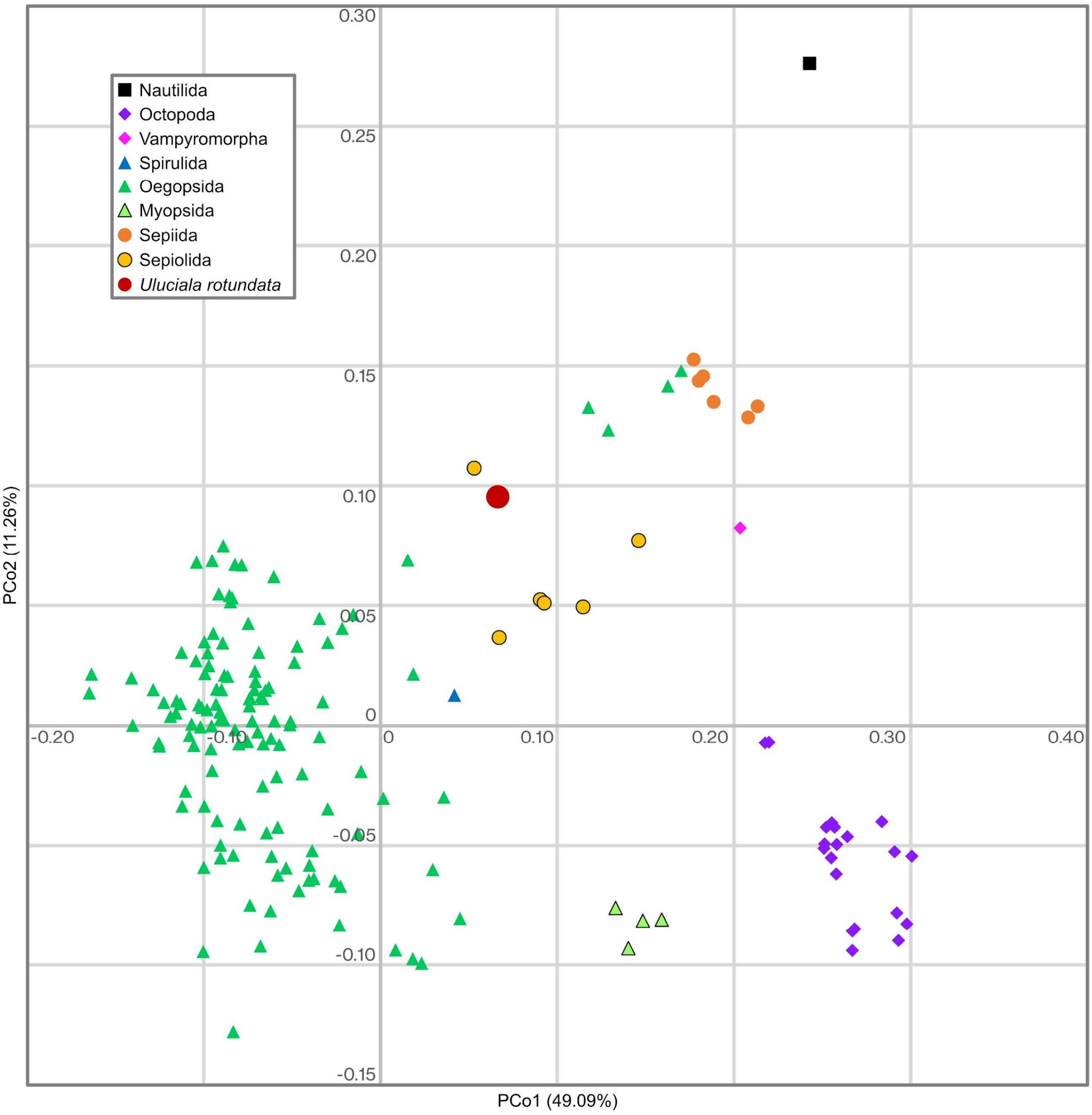

Supplementary Fig. 2. | Morphospace of the lower beak among 165 modern cephalopod species and *Uluciala rotundata* gen. et sp. nov.

Principal coordinates analysis plot showing principal coordinate 1 versus principal coordinate 2, in which the dots indicate the position of each species.

Supplementary References

1. Gower, J. C. A general coefficient of similarity and some of its properties. *Biometrics* **27**, 857–871 (1971).
2. Jereb, P. and Roper, C. F. E. *Cephalopods of the world. An annotated and illustrated catalogue of cephalopod species known to date. Volume 1. Myopsid and Oegopsid Squids. FAO Species Catalogue for Fishery Purposes. No. 4.* (Food and Agriculture Organization of the United Nations, Rome, 2005).
3. Sugiura, K. et al. The fossil lower beaks of *Uluciala rotundata. figshare* https://doi.org/10.6084/m9.figshare.28119998 (2025).
