# Supplementary material for: The oldest sepioid cephalopod from the Cretaceous discovered by Digital fossil-mining with zero-shot learning AI
Source: Commun Biol. 2026 Jan 16;9:301. doi: 10.1038/s42003-026-09519-9 (PMC12929594; doi:10.1038/s42003-026-09519-9)
Supplement: Supplementary file 3 — Description of Additional Supplementary files [file 42003_2026_9519_MOESM3_ESM.pdf]

Description of Additional Supplementary files

File name: Supplementary Data 1

Description: Details of fossil sepioid beaks and related image datasets. Institutional abbreviation: NMNS National Museum of Nature and Science, Tokyo.

File name: Supplementary Movie 1

Description: This movie shows the process of the Digital fossil-mining that incorporates a zero-shot learning AI.
