# Supplementary material for: The oldest sepioid cephalopod from the Cretaceous discovered by Digital fossil-mining with zero-shot learning AI
Source: Commun Biol. 2026 Jan 16;9:301. doi: 10.1038/s42003-026-09519-9 (PMC12929594; doi:10.1038/s42003-026-09519-9)
Supplement: Supplementary file 6 — Reporting summary [file 42003_2026_9519_MOESM6_ESM.pdf]

nature portfolio

Corresponding author(s): Yasuhiro, Iba

Last updated by author(s): Nov 30, 2025

Reporting Summary

Nature Portfolio wishes to improve the reproducibility of the work that we publish. This form provides structure for consistency and transparency in reporting. For further information on Nature Portfolio policies, see our Editorial Policies and the Editorial Policy Checklist.

Statistics

For all statistical analyses, confirm that the following items are present in the figure legend, table legend, main text, or Methods section.

n/a	Confirmed	
☒	☐	The exact sample size (*n*) for each experimental group/condition, given as a discrete number and unit of measurement
☐	☒	A statement on whether measurements were taken from distinct samples or whether the same sample was measured repeatedly
☒	☐	The statistical test(s) used AND whether they are one- or two-sided<br>*Only common tests should be described solely by name; describe more complex techniques in the Methods section.*
☒	☐	A description of all covariates tested
☒	☐	A description of any assumptions or corrections, such as tests of normality and adjustment for multiple comparisons
☒	☐	A full description of the statistical parameters including central tendency (e.g. means) or other basic estimates (e.g. regression coefficient) AND variation (e.g. standard deviation) or associated estimates of uncertainty (e.g. confidence intervals)
☒	☐	For null hypothesis testing, the test statistic (e.g. *F*, *t*, *r*) with confidence intervals, effect sizes, degrees of freedom and *P* value noted<br>*Give P values as exact values whenever suitable.*
☒	☐	For Bayesian analysis, information on the choice of priors and Markov chain Monte Carlo settings
☒	☐	For hierarchical and complex designs, identification of the appropriate level for tests and full reporting of outcomes
☒	☐	Estimates of effect sizes (e.g. Cohen's *d*, Pearson's *r*), indicating how they were calculated

Our web collection on statistics for biologists contains articles on many of the points above.

Software and code

Policy information about availability of computer code

Data collection	DEVA (https://github.com/hkchengrex/Tracking-Anything-with-DEVA), Amira v2023.2 (Thermo Fisher Scientific, Inc.), Blender v4.3.2 (Blender Foundation), and R 4.4.2 (the R Foundation)
Data analysis	The data and R code used for morphological disparity analyses are available in Figshare repository at the following link: https://doi.org/10.6084/m9.figshare.29665496. All other data supporting the findings of this study are provided in this paper.

For manuscripts utilizing custom algorithms or software that are central to the research but not yet described in published literature, software must be made available to editors and reviewers. We strongly encourage code deposition in a community repository (e.g. GitHub). See the Nature Portfolio guidelines for submitting code & software for further information.

Data

Policy information about availability of data

All manuscripts must include a data availability statement. This statement should provide the following information, where applicable:

- Accession codes, unique identifiers, or web links for publicly available datasets
- A description of any restrictions on data availability
- For clinical datasets or third party data, please ensure that the statement adheres to our policy

The image datasets of sepioid beaks discovered here and the original tomographic images of carbonate concretions are archived in the National Museum of Nature and Science, Tokyo and American Museum of Natural History. Those images are listed in Supplementary Table 1. The image datasets of sepioid beaks include 3D

models, mask data, and cross-sectional images that were cropped for annotation. These data are available from the Figshare repository at the following link: https://doi.org/10.6084/m9.figshare.28119998.

Research involving human participants, their data, or biological material

Policy information about studies with human participants or human data. See also policy information about sex, gender (identity/presentation), and sexual orientation and race, ethnicity and racism.

Reporting on sex and gender	This study is not relevant to humans.
Reporting on race, ethnicity, or other socially relevant groupings	This study is not relevant to humans.
Population characteristics	This study is not relevant to humans.
Recruitment	This study is not relevant to humans.
Ethics oversight	This study is not relevant to humans.

Note that full information on the approval of the study protocol must also be provided in the manuscript.

Field-specific reporting

Please select the one below that is the best fit for your research. If you are not sure, read the appropriate sections before making your selection.

☐ Life sciences ☐ Behavioural & social sciences ☒ Ecological, evolutionary & environmental sciences

For a reference copy of the document with all sections, see nature.com/documents/nr-reporting-summary-flat.pdf

Ecological, evolutionary & environmental sciences study design

All studies must disclose on these points even when the disclosure is negative.

Study description	Palaeontological study of earliest sepioid based on the fossil beaks.
Research sample	This study used newly discovered specimens, which were from carbonate concretions stored in American Museum of Natural History, collected from South Dakota, USA. The detailed information of studied materials, including specimen numbers, is documented in Methods and Supplementary Tables 1.
Sampling strategy	All sepioid fossils encountered in the carbonate concretions were studied. No sample size calculation was performed.
Data collection	The tomographic images of carbonate concretions were obtained by a grinding tomography system in Palaeobiology Lab of Hokkaido University. Mask data of the discovered fossils were generated by DEVA (https://github.com/hkchengrex/Tracking-Anything-with-DEVA). Reconstruction as a digital 3D model and measurements of the discovered fossils were conducted in Amira v2023.2 (Thermo Fisher Scientific, Inc.). Blender v4.3.2 (Blender Foundation) was used to display the 3D models in figures. These processes were performed by Shin Ikegami and Kanta Sugiura.
Timing and spatial scale	This study was carried out between August 2022 and May 2025. The fossils newly discovered in this study were acquired from South Dakota, USA. The detailed occurrences are documented in Methods.
Data exclusions	No data were excluded in this study.
Reproducibility	The image datasets of sepioid beaks discovered here and the original tomographic images of carbonate concretions are archived in the National Museum of Nature and Science, Tokyo and American Museum of Natural History. Those images are listed in Supplementary Table 1. The image datasets of sepioid beaks include 3D models, mask data, and cross-sectional images that were cropped for annotation. These data are available from the Figshare repository at the following link: https://doi.org/10.6084/m9.figshare.28119998. All other data supporting the findings of this study are provided in this paper.
Randomization	Randomization is not relevant since no statistical analyses were conducted for studied materials.
Blinding	Blinding is not relevant to the morphological examinations of fossils and analyses conducted herein.

Did the study involve field work? ☐ Yes ☒ No

Reporting for specific materials, systems and methods

We require information from authors about some types of materials, experimental systems and methods used in many studies. Here, indicate whether each material, system or method listed is relevant to your study. If you are not sure if a list item applies to your research, read the appropriate section before selecting a response.

Materials & experimental systems

n/a	Involved in the study
☒	☐ Antibodies
☒	☐ Eukaryotic cell lines
☐	☒ Palaeontology and archaeology
☒	☐ Animals and other organisms
☒	☐ Clinical data
☒	☐ Dual use research of concern
☒	☐ Plants

Methods

n/a	Involved in the study
☒	☐ ChIP-seq
☒	☐ Flow cytometry
☒	☐ MRI-based neuroimaging

Palaeontology and Archaeology

Specimen provenance

A statement on the provenance of the specimens is provided in Methods and Supplementary Table 1.

Specimen deposition

The image datasets of sepioid beaks discovered here and the original tomographic images of carbonate concretions are archived in the National Museum of Nature and Science, Tokyo and American Museum of Natural History. Those images are listed in Supplementary Table 1. The image datasets of sepioid beak include 3D models, mask data, and cross-sectional images that were cropped for annotation. These data are available from the Figshare repository at the following link: https://doi.org/10.6084/m9.figshare.28119998.

Dating methods

The age of samples was determined based on published biostratigraphic data from the collection site.

☐ Tick this box to confirm that the raw and calibrated dates are available in the paper or in Supplementary Information.

Ethics oversight

Hokkaido University

Note that full information on the approval of the study protocol must also be provided in the manuscript.

Plants

Seed stocks

This study is not relevant to plants.

Novel plant genotypes

This study is not relevant to plants.

Authentication

This study is not relevant to plants.
